# Cytotoxic Effects of Various Mineral Trioxide Aggregate Formulations, Calcium-Enriched Mixture and a New Cement on Human Pulp Stem Cells

**Published:** 2014-10-07

**Authors:** Zahra Jaberiansari, Seddigheh Naderi, Fahimeh Sadat Tabatabaei

**Affiliations:** a *Department of Operative Dentistry, Dental School, Shahid Beheshti University of Medical Sciences, Tehran, Iran;*; b *Dentist, Dental School, Shahid Beheshti University of Medical Sciences, Tehran, Iran;*; c *Department of Dental Materials, Dental School, Shahid Beheshti University of Medical Sciences, Tehran, Iran*

**Keywords:** Angelus MTA, Calcium-Enriched Mixture, CEM Cement, Cytotoxicity, Dental Pulp Cells, Mineral Trioxide Aggregate, MTT Assay, ProRoot MTA, Root MTA, Stem Cells

## Abstract

**Introduction:** This *in vitro* study compared the cytotoxic effects of three commercially available MTA formulations naming ProRoot MTA (PMTA), Angelus MTA (AMTA), and Root MTA (RMTA), with calcium-enriched mixture (CEM) cement and a new nanohybrid MTA (NMTA) on human dental pulp stem cells (DPSC). **Methods and Materials:** Four disc-shaped specimens of each material were prepared. After completion of setting, 2 different (neat and 1/2) elutes of the test materials were made. Then in each cavity of a 96-well plate, 3000 cells were seeded and incubated in a humidified incubator with 5% CO_2_ and 95% air at 37^°^C for 24 h. After this period, the culture medium of each well was replaced with 200 μL of test material elutes. Plain culture medium was used as the negative control and distilled water as the positive control group. Cell viability was assessed using 2, 5-diphenyl-SH-tetrazelium bromide colorimetric assay, *aka* Mosmann’s tetrazolium toxicity (MTT) assay, at three time intervals (24, 48, and 72 h after mixing). Data were analyzed using the ANOVA and Tukey’s post hoc test (*P*=0.05). **Results: **After 24 h, the viability of cells in neat concentration had no significant differences (*P*>0.05) except for the NMTA. However, CEM and AMTA, at 1/2 concentration exerted significant proliferative effects on cells. At 48 and 72-h intervals, significant proliferation of DPSCs was seen in all samples, except for the NMTA which exerted toxic effects on cells. **Conclusion: **All of the three commercial MTAs and CEM cement showed comparative biocompatibility. However, NMTA had cytotoxic effects on DPSCs at all the time intervals.

## Introduction

Direct pulp capping (DPC) refers to sealing of the exposed vital pulp by using biocompatible and bioregenerative dental materials in order to enhance the formation of tertiary dentinal bridge and preserve the pulp vitality [1]. Following DPC, primary odontoblasts can produce reactive dentine or if they are dead they are replaced by the secondary odontoblast-like cells differentiated from dental pulp stem cells (DPSCs) which deposit the reparative dentine [2]. DPC was first introduced by Philip Pfaf in 1756 by placing a tiny piece of gold foil over the exposed dental pulp [1]. In 1923, Davis suggested using a mixture of zinc sulfate for DPC [3]. Calcium hydroxide (CaOH2) or CH was first used by Herman in 1930 for DPC with successful outcomes. Since early 1940s, CH has been the most commonly used material for DPC [4, 5]. Despite numerous advantages, CH has some shortcomings namely high porosity, inadequate bond to dentin, chemical degradation and dissolution over time and consequent microleakage. Thus, it is not optimal for pulp capping [6].

Mineral trioxide aggregate (MTA) was first developed in Loma Linda University in 1993 as a root-end sealing materilal [7]. ProRoot MTA (PMTA; Dentsply, Tulsa Dental, Tulsa, OK, USA) is known as an optimal material for pulp capping due to several advantages such as high sealing ability, prevention of bacterial leakage, high biocompatibility and no disintegration over time. It owns a primary pH of 12.5, has an easy application and induces cementogenesis, osteogenesis [8, 9] and dentinogenesis [10]. Based on the literature, when used for DPC, MTA shows greater interaction with pulp compared to CH [11]. During the last years many formulations of MTA have been introduced to the market.

Angelus MTA (AMTA; Angelus, Londrina, Parana, Brazil) is comprised of 80% Portland cement and 20% bismuth oxide. Based on the literature, AMTA has slightly higher pH and greater calcium release than PMTA [8]. Moreover, chemical composition and crystalline structure of this type of MTA have been investigated in several studies and lower amount of bismuth oxide has been reported in AMTA compared to PMTA [12].

Root MTA (RMTA; Lotfi research group, Tabriz, Iran) is mainly comprised of synthetic crystals. It’s important constituents include calcium silicate and aluminum silicate [13, 14]. The scientific and industrial research organization of Iran qualitatively analyzed this material and compared it with PMTA and subsequently confirmed their similarity [15].

In 2006, calcium-enriched mixture (CEM) cement (BioniqueDent, Tehran, Iran) was introduced. CEM cement is a white powder made of hydrophilic materials that sets in presence of water-based liquids. The hydration of powder forms a colloidal gel that hardens in less than an hour and forms hydroxyapatite. Its composition includes calcium oxide, calcium phosphate, calcium silicate and calcium sulfate. However, its chemical composition is different from PMTA [16]. The properties of CEM cement have been investigated in several studies [17-19].

Beside the different cements available in the dental market, the newer formulations of MTA are still under investigation. One of them is an experimental cement named as nanohybrid MTA (NMTA) containing three different nanoparticles (based on the inventor’s claim).

Almost all of the in vitro studies considering the bioavailability of these cements, have evaluated their effects on endothelial cells, cancer cells, blood cells, odontoblast-like cells, human gingival fibroblasts, L929 fibroblasts, and periodontal ligament fibroblasts [15, 20-27]. This study aimed to compare the cytotoxicity of different MTA formulations, *i.e*. PMTA, RMTA, AMTA and CEM cement which are available in Iranian market to that of the new NMTA on human DPSCs.

## Methods and Materials

***Sample preparation:***

In this in vitro study, PMTA (ProRoot MTA, Dentsply, Tulsa Dental, Tulsa, OK, USA), AMTA (Angelus MTA, Londrina, Paraná, Brazil), RMTA (Root MTA Lotfi research group, Tabriz, Iran), CEM cement (BioniqueDent, Tehran, Iran) and an experimental cement (NMTA) were separately mixed under sterile conditions on a glass slab exactly according to manufacturers’ instructions and transferred into wells with 2 cm diameter and 3 mm height in a 24-well plate (SPL Life Science, Gyeonggi-do, South Korea). The plate was stored in an incubator (Memmert, Schwabach, Germany) at 37°C and 95% humidity for 24 h to allow complete setting of the materials. The plate was then removed from the incubator and serum and antibiotic free Dulbecco's modified Eagle's medium (DMEM, Gibco, Grand Island, NY, USA) was added to the wells and incubated for 24 h to obtain elute of materials [28]. After 24 h, elutes were sterilized using 0.2 µm filter. To observe a dose-response relationship, elutes were diluted with DMEM to achieve 2 concentrations of each elute (neat and 1/2) that were then tested for cytotoxicity as described. Finally 1% antibiotic (Gibco, Grand Island, NY, USA) and 10% fetal bovine serum (FBS, Gibco, Grand Island, NY, USA) were added to the elutes.

***Cell cultures:***

DPSCs previously detected by flow cytometry and cell differentiation were procured from the Molecular and Cell Biology Laboratory of Shahid Beheshti Dental School. Cells were transferred from the nitrogen tank to 37^°^C water bath. After defrosting, culture medium was gradually added to cells and after centrifuging and extraction of the previous medium, cell suspension was transferred to a flask (SPL, Gyeonggi-do, South Korea) containing DMEM, 10% FBS and 1% antibiotic and incubated at 37^°^C with 5% CO_2_ and 95% humidity. Fourth passage of cells, after reaching 80% confluence in the flask, were gradually separated from the bottom of the dish using 0.25% trypsin (Sigma-Aldrich, St. Louis, MO, USA) and counted using the Neubauer chamber. Then 3000 cells/200 μL culture medium were transferred to each well of 96-well plate. Six plates were prepared. Peripheral wells of the plates only contained plain culture medium and were considered blank. Plates were transferred to an incubator (in a humidified atmosphere of air and 5% CO_2_, at 37^°^C). After 24 h, the culture media were replaced with 200 μL of the prepared elutes or control media, and the cells were stored in an incubator for 24, 48 and 72 h. Two columns (neat and 1/2 concentrations) of the 96-well plate *(n=*6*)* were allocated to each of the understudy materials. Sterile distilled water which is toxic for cells was used as the positive control and DMEM containing 10% FBS and 1% antibiotic was considered as the negative control group.

***Cytotoxicity assay:***

Cytotoxicity of the materials was assessed using dimethyl-thiazole-diphenyl tetrazolium bromide assay or Mosmann’s Tetrazolium Toxicity (MTT) assay (Sigma Aldrich, St. Louis, MO, USA). At 24, 48 and 72 h, culture medium was rinsed off by phosphate-buffered saline (PBS) (Gibco BRL, Grand Island, NY, USA) for washing. Then MTT solution was prepared at 5 mg/mL concentration and diluted with FBS-free culture medium in a 1/10 ratio; 100 μL of this solution was added to each well. The plates were then stored in an incubator at 37^°^C, 98% humidity and 5% CO_2_. During this time period, viable cells converted the soluble yellow MTT salt into insoluble purple formazan crystals using mitochondrial succinate dehydrogenase enzyme. After 3 h of incubation, the culture medium on the top was gradually extracted and 100 μL of dimethyl sulfoxide (DMSO, Gibco BRL, Grand Island, NY, USA) was added to each well to dissolve formazan crystals. The resultant discoloration had a direct correlation with the metabolic activity of cells and was measured by ELISA reader (Anthos 2020, Wales, Australia) at 570 nm wavelength with 650 nm filter. All assays were repeated three times to guarantee their reproducibility.

**Figure 1:**
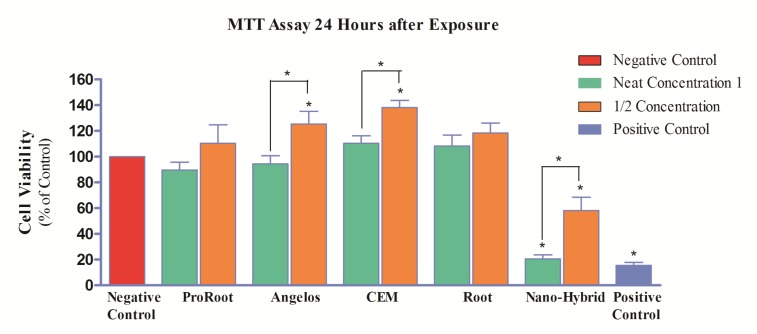
Percentage of pulp stem cell viability (mg/mL) following exposure to different cements after 24 h (*=Significant difference)

***Statistical analysis:***

All tests were repeated 3 times. Based on the ISO 10993-5, the percentage of cell viability was obtained by dividing the mean absorbance of each group by the mean absorbance of the negative control group multiplied by 100. The percentage of cell viability over 90% indicated non-toxicity, while the amounts between 60-90%, 30-60% and less than 30%, indicated mild, moderate and severe toxicity, respectively. Data were analyzed using GraphPad Prism software (GraphPad Software, San Diego, CA, USA), the ANOVA and post-hoc Tukey’s tests. The level of significance was set at 0.05.

## Results

***After 24 h of exposure:***

As seen in Figure 1, the difference in cell viability between groups exposed to neat concentration of the three MTA cements, CEM cement and negative control group was not significant (*P*>0.05). However, at concentration of 1/2, proliferation of cells significantly increased in CEM cement and AMTA groups compared to the negative control group (*P*<0.05).

The viability of cells exposed to neat concentration of NMTA was significantly low compared to the negative control group (*P*<0.05) which is indicative of severe toxicity. There were also statistically significant differences (*P*<0.05) between cell viability of this material in concentration of 1/2 compared to the negative control group (moderate cytotoxicity).

***After 48 h of exposure:***

**Figure 2:**
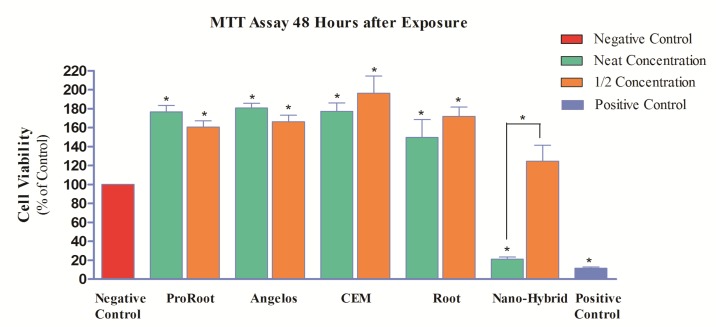
Percentage of pulp stem cell viability (mg/mL) following exposure to different cements after 48 h (*=Significant difference)

A significant increase in cell proliferation was seen in all groups exposed to all cements compared to the negative control group (*P*<0.05) (Figure 2).

The percentage of viability of cells exposed to NMTA at neat concentration was 21%; which showed 79% reduction compared to the negative control group and this reduction was statistically significant (*P*<0.05). Thus, at 48 h, the neat concentration of this material was still severely toxic. However, this material in concentration of 1/2 showed 24% increase compared to the negative control group which was not statistically significant (*P*>0.05). This concentration was no longer cytotoxic after 48 h.

***After 72 h of exposure:***

**Figure 3:**
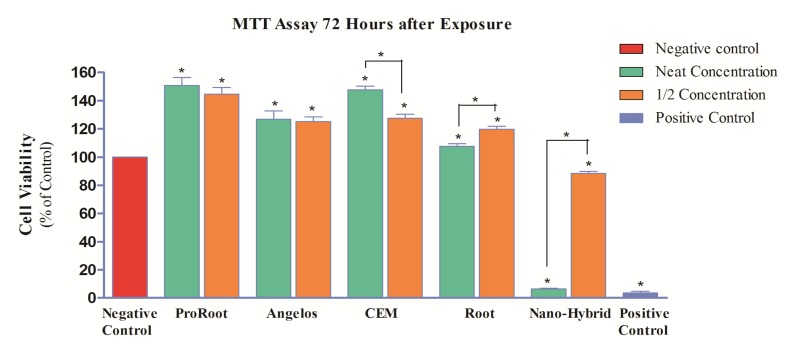
Percentage of pulp stem cell viability (mg/mL) following exposure to different cements after 72 h (*=Significant difference)

In the third MTT assay (72 h), a significant increase in cell proliferation was noted in all groups, compared to the negative control group (*P*<0.05). However, in CEM cement group in concentration of 1/2 a significant reduction by 20.1% was noted (*P*<0.05). Also, the difference between the two concentrations of RMTA was statistically significant (*P*<0.05) and cell viability at concentration of 1/2 was higher by 12.1% compared to its neat concentration (Figure 3). Neat NMTA was still severely cytotoxic at 72 h and 1/2 concentration of this material had moderate cytotoxicity after 72 h.

***The effect of time on the viability of cells exposed to different cements:***

In all MTA groups and CEM cement, a significant time-dependent exponential increase in cell proliferation and viability was seen at neat and 1/2 concentrations (*P*<0.05). NMTA at 1/2 concentration caused a time-dependent increase in cell proliferation and viability while its neat concentration maintained its cytotoxicity over time.

***Cements:***

In all MTA groups and CEM cement, a significant time-dependent exponential increase in cell proliferation and viability was seen at neat and 1/2 concentrations (*P*<0.05). NMTA at 1/2 concentration caused a time-dependent increase in cell proliferation and viability while its neat concentration maintained its cytotoxicity over time.

## Discussion

This study compared the cytotoxic effect of four MTA formulations and CEM cement on DPSCs during different time intervals. Preservation of pulp vitality during restorative treatments is an ideal goal that prevents change of the treatment plan towards root canal therapy and increases the survival of the tooth. Thus, using a bioactive material that can optimally seal the pulp exposure site is very important [2].

MTA has successfully served this purpose in the recent years. MTA is a bioactive material that is hard tissue inductive, hard tissue conductive and biocompatible [29]. New MTA formulations have been introduced and need to undergo *in vitro *biocompatibility tests as the primary assessment [30]. Selection of the type of laboratory assay depends on the chemical composition of the test materials. Considering the hydrophilicity of MTA and its released ions, evaluation of the activity of intracellular enzymes using MTT assay seems to be more accurate than the membrane permeability tests [21]. Selection of the type of cells in this assay should be based on the application of the material. Since MTA cement as a DPC agent will be in close contact with dental pulp, assessment of its behavior and effect on DPSCs is of utmost importance. However, to date, limited studies have used these cells [23, 31, 32].

Overall, freshly mixed materials have higher cytotoxicity due to the release of some materials during their setting. After completion of setting, the material becomes structurally more stable and its primary cytotoxicity decreases [29]. Therefore, in the present study, cells were exposed to elutes of set materials, like some other similar studies [15, 33]. However, considering the fact that in the clinical setting, these materials are not applied in the set form, future studies are recommended to evaluate a group of samples receiving freshly mixed materials. In the present study, this issue could not be evaluated due to the limited amount of materials.

One advantage of eluting the materials is their easy sterilization. Moreover, the effect of released materials on cells can be evaluated and different concentrations of elutes can be prepared and the effect of dosage can be investigated as well [21]. In this study, based on the 10993-5 ISO standard, specific amount of culture medium was added to discs and elutes in concentration of 1/2 were also prepared. A significant difference was found between the two concentrations of NMTA, AMTA, CEM cement and RMTA at different time intervals (P<0.05). In other words, the proliferative effect of all materials (except for CEM cement) increased by decreasing their concentration which shows a dose-dependent cytotoxicity. In CEM cement group, by decreasing the concentration, an increase in cell proliferation was noted at 24 h and 48 h but the proliferation of cells at 24 h and 48 h in presence of 1/2 concentration of the cement was very high; at 72 h, the cells entered into a growth decline phase due to their high density and all available adhesion surfaces in wells being occupied. Therefore, the lower cell viability observed at 1/2 concentration of CEM cement was not due to its higher cytotoxicity. In the study by Ghoddusi *et al*. [18], neat, 1/2, 1/10 and 1/100 concentrations of CEM cement and PMTA were used and by decreasing the concentration, cytotoxicity decreased as well.

In this study, a significant difference was noted between different time points at neat and 1/2 concentrations of the materials (except for the neat NMTA) (P<0.05). In other words, cell proliferation and viability were time-dependent and followed this order: 72 h> 48 h> 24 h. Our findings in this regard were in agreement with those of Mozayeni et al.’s [24], (evaluation of cytotoxicity at 1h, 24 h and 7 days), Koulaouzidou *et al.’s* [34]

(evaluation of cytotoxicity at 24 h and 72 h) and De Deus *et al.’s* [20] (evaluation of cytotoxicity at 24 h, 48 h and 72 h). Also, no significant difference was noted between different MTA cements at different concentrations and various time points. Sharifian *et al*. [25] showed similar effects of fresh, 4-h, one-day and 7-day set PMTA, RMTA and Portland cement on L929 mouse fibroblasts. However, in a study by Javaheri *et al*. [15], on the cytotoxicity of PMTA, RMTA and Portland Cement, the highest cytotoxicity belonged to RMTA and the lowest to PMTA. The results of their study which were reported only at 24 h were in contrast to our findings at 24 h; which may be due to the different types of target cells (stem cells in the present study and peripheral blood mononuclear cells in the other study), method of cytotoxicity assessment (MTT assay in the present study and viable cell counting at the bottom of plates in the other study) and also direct exposure of cells to materials and using one concentration of test materials in the other studies.

In a study by Ghoddusi *et al.* [18], PMTA and CEM cement showed similar cytotoxic effects on L929 mouse fibroblasts after 24, 48 and 72 h; which is in agreement with our results. However, Mozayeni et al. [24] compared the cytotoxicity of CEM cement and PMTA at 1, 24 and 7 days and found that the cytotoxicity of CEM cement was higher than that of PMTA [24]. Such conflicting results may be attributed to the different type of target cells used (L929 mouse fibroblasts).

Our results also confirmed those of Koulaouzidou *et al.’s* [34] who compared the cytotoxicity of AMTA and PMTA against human fibroblasts and mouse dental pulp cells at 24 and 72 h using XTT assay and found similar cytotoxicity of the test materials. Furthermore, De Deus et al. [20] used human endothelial cells to assess the cytotoxic effects of AMTA, PMTA and Portland cement at 24, 48 and 72 h and detected no significant differences among them.

Our findings revealed that neat concentration of NMTA had significant cytotoxicity at all three time intervals (P<0.05). However, due to the presence of numerous confounding factors in the oral environment, the results of this in vitro study may not be completely generalized to the clinical setting and clinical trial are required.

## Conclusion

Cytotoxicity of the understudy materials was dependent on their dosage and exposure time. The lower concentrations at longer exposure times showed more favorable results. All evaluated types of MTA cements in this study had similar effects on human DPSCs. Within the limitations of this *in vitro* study, the new formulation of MTA cement was toxic for DPSCs.
